# Optimizing imaging resolution in brain MRI: understanding the impact of technical factors

**DOI:** 10.25122/jml-2022-0212

**Published:** 2023-06

**Authors:** Shapol Yousif Ahmed, Fatiheea Fatihalla Hassan

**Affiliations:** 1 Department of Basic Sciences, College of Medicine, Hawler Medical University, Erbil, Iraq

**Keywords:** Imaging, Brain, MRI, Brain MRI

## Abstract

Magnetic resonance imaging (MRI) exams are essential for diagnostic procedures, but their lengthy duration and associated costs limit their accessibility. Shorter scan times would reduce expenses and allow for more MRI exams, expanding the range of diagnostic procedures. This study investigated technical factors that could decrease scan time without compromising image quality, including field-of-view (FOV), phase field of view, phase oversampling, cross-talk, brain MRI imaging resolution, and scan time. Data were collected from September 2021 to June 2022. All patients underwent brain scans in the transverse plane following a standardized protocol using a 1.5-tesla Siemens Avanto MRI scanner. The protocol employed T2-weighted Turbo Spin Echo imaging. Twenty-four cases were included in this study. Initially, all participants underwent brain MRI scans using the original protocols with axial sections. The results indicated that altering the FOV phase and phase oversampling significantly affected the scan time, whereas other factors did not have a direct impact. The original protocol had a scan time of 3.47 minutes with a FOV of 230 mm, 90% FOV phase, and 0% phase oversampling. After implementing the modified protocol, the scan time was reduced to 2.18 minutes with a FOV of 217 mm and 93.98% phase oversampling of 13.96%. Statistical analysis confirmed the high significance of FOV phase and phase oversampling in reducing scan time. By optimizing these technical factors, MRI exams can be performed more efficiently, resulting in shorter scan times and potentially reducing costs. This would enhance patient comfort and enable a greater number of MRI exams, facilitating a more comprehensive range of diagnostic procedures.

## INTRODUCTION

Magnetic resonance imaging (MRI) is a well-established yet time-consuming radiological imaging method. Efforts have been made to minimize image acquisition durations due to increased demand, economic pressures, restricted patient comfort, and the need to enhance image resolution without lengthening image acquisition [1]. Medical image processing methods use techniques of imaging to offer a platform for medical specialists and researchers to investigate and evaluate the internal activities of the brain and anatomical structures. Researchers and medical practitioners use physiological or functional imaging and anatomical or structural imaging to look at the structure and functions of the brain without surgery [2]. However, achieving high-resolution imaging of the human body presents challenges related to signal-to-noise ratio (SNR), scan duration, and resolution. Balancing these factors often requires compromises [3]. Increasing the resolution and SNR can lead to longer scan times, as the voxel volume is proportional to the SNR of the MR data and the resulting image.

Similar to the regulation of radiation dosage in computed tomography (CT) examinations, controlling the duration of MRI exams has become an important consideration. Shorter MRI exams offer cost advantages and the potential to serve a larger number of patients, thereby enabling a broader range of diagnostic procedures [4, 5].

The main aim of the study was to achieve optimal imaging resolution, which is directly influenced by technical factors affecting scan time and image quality. The study was conducted at the Department of Radiology at Erbil teaching hospital and investigated technical factors such as field-of-view, phase field of view, phase oversampling, cross-talk (distance factor), slice thickness, and slices of brain MRI imaging resolution and scan time.

## MATERIAL AND METHODS

### Study setting and participants

This study was conducted at the Department of Radiology from Erbil teaching hospital, Erbil, Iraq. Data were collected from September 2021 to June 2022. The study included patients scheduled for a brain scan in the transverse plane (axial section) using the standardized protocol without modifying the original factors. Patients with orthodontics, metal tooth implants, and mechanical heart valves were excluded from the study. Patients with psychiatric conditions and those who were unconscious or too fatigued to tolerate the lengthy scanning procedure were also excluded. A total of 24 adult patients, ranging in age from 23 to 45 years, participated in the study, with 14 females and the remaining males.

### MRI scanning protocol

All scans were performed on a 1.5-tesla Siemens Avanto MRI system using the standard quadrature birdcage transmit/receive head coil. The imaging protocol used a T2-weighted fast spin-echo in the transverse plane. The T2-weighted sequence is a fundamental pulse sequence in MRI that highlights variations in the T2 relaxation time among different tissues. The raw data required for image reconstruction were captured in multiple successive passes, providing benefits such as shorter effective echo time, narrower bandwidth, reduced T2 decay, and fewer artifacts. To achieve a balance between scan time reduction and optimal image spatial resolution, several factors were adjusted. The field-of-view (FOV) was reduced to 217 mm, compared to the standardized FOV of 230 mm. The FOV phase was 93.88% instead of the standardized FOV phase of 90.6%. Additionally, phase oversampling was increased to enhance the signal-to-noise ratio.

## STATISTICAL ANALYSIS

Data was entered and analyzed using the Statistical Package for Social Sciences (SPSS version 28). Descriptive statistics, including mean and standard deviation (SD), were used to summarize and describe the numerical variables, such as FOV and FOV phase. To assess the significance of differences between groups, the t-test for two independent samples was employed. Additionally, the one-way analysis of variance (ANOVA) was utilized to compare means across more than two samples. In particular, the mean of the FOV phase was compared with scan time using ANOVA. A P-value of less than 0.01 indicated a highly significant difference.

## RESULT

This study aimed to investigate the impact of technical factors on scan time and imaging resolution in brain MRI. A total of 24 cases were included, and all participants underwent brain MRI scans using the original protocols with axial sections. The effects of changing parameters were evaluated, including field-of-view (FOV), phase field of view, phase oversampling, cross-talk, slice thickness, and several slices. The statistical analysis of physical parameters is presented in Table 1. The average slice thickness was 4.98 mm, and the average number of slices was 17.50. The relationship between parameters and scan time is presented using t-test and F-test results. The results of the one-way ANOVA show that the FOV phase (p<0.001) and sampling (p<0.001) had a significant impact on scan time (Table 2).

**Table 1:** Statistical analysis of physical parameters

	slice thick	no. slice	FOV	FOV phase	cross-talk	sampling	SNR	scan time	Time second
**N**	24	24	24	24	24	24	24	24	24
**Mean**	4.98	17.50	217.67	93.888	38.79	13.96	0.9517	2.1638	138.04
**Median**	5.00	17.00	216.00	95.300	38.50	12.50	0.9650	2.1700	137.00
**Mode**	5	17	216	95.3	30	17	1.00	2.22	142
**Std**. **Deviation**	0.102	0.933	6.742	3.3861	6.129	10.461	0.07883	0.17340	12.682

**Table 2:** The relation between parameters and scan time

	B	t-test	p-Value	F-test One-Way ANOVA P-Value	Adjusted R Square
**(Constant)**	-7.167	-0.392	0.700	471.904 <0.001 (HS)	0.988
**FOV**	0.020	0.369	0.716		
**FOV phase**	1.359	13.418	<0.001		
**Cross-talk**	-0.065	-1.282	0.215		
**Sampling**	1.128	38.644	<0.001		
Time second = -7.167+0.020 FOV + 1.359 FOV phase - 0.065 cross-talk + 1.128 sampling					

**Figure 1:**
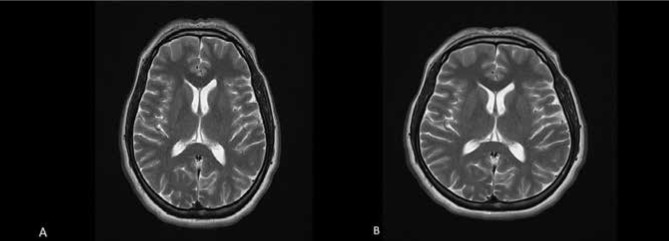
Comparison of brain MRI T2-weighted 2D TSE axial section images in the original protocol and after changing parameters. A) Original protocol: scan time (TA) of 3.47 minutes, FOV of 230 mm, 90% FOV phase, and 0% phase oversampling. B) Modified protocol: scan time (TA) of 2.18 minutes, FOV of 217 mm, and 93.98% phase oversampling of 13.96%.

**Figure 2:**
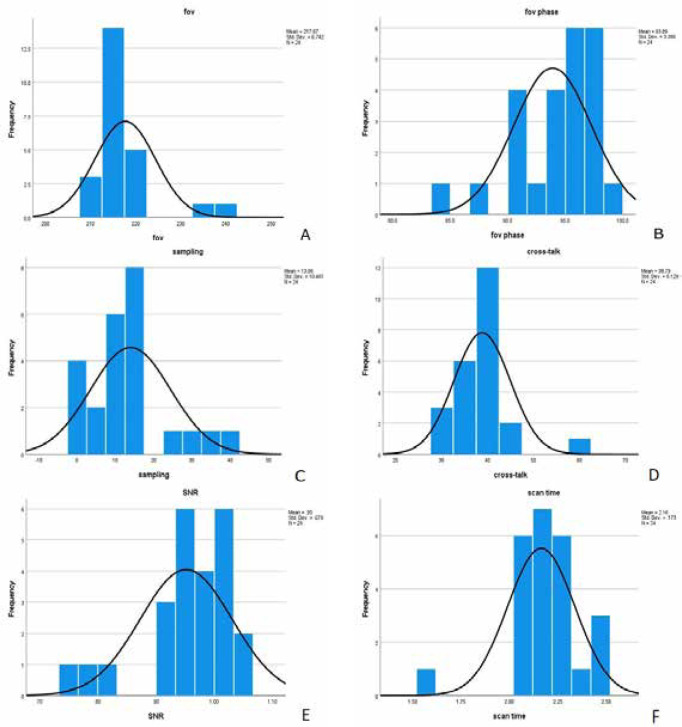
Frequency and parameters analyzed after modifying the standard T2-TRS-tra protocol. A) Mean FOV = 217 mm; B) FOV phase = 94%; C) Phase oversampling = 14%; D) Cross-talk = 39%; E) Signal-to-Noise Ratio (SNR) = 0.9517 tse; F) Scan time = 2.17 minutes.

The T2-weighted 2D TSE axial section images of the same case obtained using the original protocol and the modified protocol with changed parameters are presented in Figure 1. The original protocol had a scan time of 3.47 minutes, a FOV of 230 mm, a FOV phase of 90%, and 0% phase oversampling (Figure 1 A). The brain MRI image, after implementing the modified protocol, resulted in a reduced scan time of 2.18 minutes, a FOV of 217 mm, and 93.98% phase oversampling of 13.96% (Figure 1 B). Figure 2 (A-F) provides a graphical representation of the frequency and parameters analyzed in the study.

## DISCUSSION

In this study, we aimed to investigate the impact of changing various technical factors on the scan time and imaging resolution of brain MRI. Our results revealed important insights into the relationship between these factors and the scan parameters. First, we found no relationship between FOV and scan time. Decreasing the FOV had no direct effect on scan time, but spatial resolution increased and increased the likelihood of aliasing. The mean FOV of 217 mm used in our study did not exhibit any aliasing artefacts (p=0.716). Another parameter that influenced the resolution of MRI images was the slice thickness. As the slice thickness increases, other tissues are included in the slice, leading to image blurring and reduced image quality due to partial volume effects [6]. In our study, the average slice thickness was 4.98 mm, higher than the optimal thickness reported in other studies for diagnostic and therapeutic purposes in brain MRI, typically less than 1 mm [7,8].

In a study by K Wengler *et al*. on the reproducibility of MRI protocols, the average number of slices ranged from 8 to 16 [9]. Our study examined approximately 18 slices, which is a relatively high number compared to other studies [10].

Cross-talk is a phenomenon that occurs due to the interference between adjacent slices in MRI imaging, and it is primarily caused by the rectangular shape of the slices generated by the RF pulses. In our study, the average cross-talk value was 38.79, with no significant effects on scan time (p-value=0.215). Previous research has indicated that modifying the angle and rotation of the patient can help reduce cross-talk and improve image quality [11]. Nevertheless, more emphasis should be paid to reducing the effect of cross-talk artefacts and increasing the signal intensity uniformly.

The field of view (FOV) in MRI determines the specific area of the patient's body that will be captured in the image. It is selected prior to the scanning process and can be adjusted based on the desired imaging requirements. In certain scenarios, it can be advantageous to reduce the FOV along one axis, typically expressed in centimeters or millimeters. On the other hand, the field of view phase is a parameter set by the technician and represents a subset of the overall FOV. It is a valuable tool in reducing scanning time and eliminating unnecessary areas of the body from the final image. By optimizing the field of view and phase FOV, imaging time can be minimized without compromising the diagnostic quality of the images [12]. According to brain MRI studies, utilizing a FOV of 230 mm or less is common to achieve high spatial resolution on the screen. This is because the FOV increases with the number of pixels, improving the signal-to-noise ratio (SNR) and producing smoother and more detailed images [13]. Based on the results, the average FOV in this study was 217.67, which corresponds to the standard range for brain MRI and is consistent with other studies [14]. Given that the FOV phase refers to the size of the field of view in the phase direction and is usually expressed as a percentage of the base field. If we express the FOV phase as 100%, the length and width of the image will be equal, and the resulting image will be square, but if it is less than 100%, then a rectangular FOV will be created. Our study found a FOV phase value of 93.88, which aligns with most studies using a non-100% FOV phase [15].

Phase oversampling, with a highly significant p-value of <0.01, shows a linear relationship with scan time. Increasing phase oversampling enhances the scan time and SNR, which partially compensates for the added time. Because the extra phase encoding stages enhance the signal-to-noise ratio, this approach does not always result in a loss of image quality (enhancing the phase oversampling increases the number of phase-encoding steps and FOV in the phase direction).

The FOV phase approach obtains a separate scanned area in the phase encoding directions. It implies that data is collected with fewer measurement lines, resulting in a shorter scan time. Since there are fewer rows than columns, the result is a rectangular image, providing the name of the method. The SNR is reduced when the FOV is reduced in the phase encoding direction, while the spatial resolution is always maintained. A fold-over artefact will develop if the item is bigger in this phase direction than in the FOV phase [16, 17]. In our study, the FOV phase showed a highly significant p-value of <0.01, indicating that increasing the FOV phase also increases scan time.

The sampling obtained in our study was 13.96, consistent with other studies where sample sizes typically range from 10 to 20. Small volumes usually reduce the reproducibility of studies [18].

Signal-to-noise ratio (SNR) is an important quantity used for performance evaluation and is often used for image evaluation, contrast enhancement measurements, pulse sequences, and radio frequency (RF) coils. The results showed that the average SNR in this study was 0.951, while in most other studies, this value is greater than one, and the standard range of N30 is -18 db [19-21].Examining the duration of the scan showed that it took about 2-3 minutes, which is consistent with the duration of scans in other studies [22].

Slice thickness (ST) plays a significant role in sequence SNR, with small changes in ST resulting in significant SNR changes that can be traded off for in-plane resolution and scan time. Increasing ST improves voxel size and enhances SNR, but it reduces spatial resolution and increases partial volume effects. The mean slice thickness in this study was 5 mm, resulting in a good SNR and acceptable spatial resolution.

Scan time refers to the duration of the imaging process and is influenced by various factors. Increasing resolution reduces the signal, necessitating time-increasing parameters to acquire the desired signal. Several methods can be employed to reduce scan duration while maintaining image quality. A previous study by Sartoretti *et al*. [1] explored parameters to minimize the time required for MRI scans across six anatomical regions, including the brain. It demonstrated that high-resolution MRI could be achieved with minimal procedure time. Overall, our brain MRI scans, after modifying several factors, maintained spatial resolution with high SNR, without noise or artifacts, and decreased scan time.

## CONCLUSION

MRI imaging is a valuable but time-consuming technique in radiology. Given the high demand and the need for efficient patient care, there have been efforts to reduce scan time by modifying certain factors. Our study focused on evaluating the impact of changing specific factors on scan time. We found that altering the field of view (FOV) phase and implementing phase oversampling significantly reduced scan time, while other factors did not directly influence the scan duration. These modifications can be implemented without compromising the main objective of obtaining high-resolution, high-quality imaging. By optimizing scan time, healthcare facilities can minimize costs and accommodate more patients needing this imaging modality.

## Personal thanks

The authors would like to express their gratitude to all the staff members of the Department of Radiology at Erbil Teaching Hospital, Erbil, Iraq, for their valuable cooperation and the use of their facilities, which significantly contributed to the successful completion of this research.
